# Long-term efficacy of neural circuit blockade for treating blepharospasm: a retrospective case series study

**DOI:** 10.3389/fneur.2026.1847839

**Published:** 2026-06-24

**Authors:** Gang Liu, Qiangying Guo, Jie Xiang, Zhen Xu, Suying Chen, Yanling Xue, Zhitao Liu, Huixia Jin, Shangyi Yu, Ting Jiao, Xia Chen, Zhenzhen Liu, Xiangying Xi, Yanhong Li, Lili Shang, Jiao Du, Xianzhong Liu, Shihui Wei

**Affiliations:** 1 Meige Syndrome Center, The Third People’s Hospital of Henan Province, Zhengzhou, China; 2 Senior Department of Ophthalmology, The Chinese People’s Liberation Army General Hospital, Beijing, China

**Keywords:** blepharospasm, long-term efficacy, Meige syndrome, neural circuit blockade, retrospective study

## Abstract

**Background:**

Blepharospasm is a common symptom of Meige syndrome and often leads to functional blindness. Although current treatment options, such as oral medications, botulinum toxin injections, resection of periocular muscles, and deep brain stimulation, are available, no definitive method effectively eliminates blepharospasm.

**Objective:**

To address this unmet need, we developed a novel approach—neural circuit blockade (NCB). This study aimed to analyse its long-term efficacy for blepharospasm.

**Methods:**

NCB eliminates trigeminal nerve stimulation by facial expression muscles, reducing the production and input of sensory information, weakening feedback-based and plastic motor information functions, decreasing orbicularis oculi muscle tension, and alleviating blepharospasm. A retrospective analysis was conducted on 570 patients who underwent NCB treatment, with a median postoperative follow-up of 51 months. Therapeutic efficacy was evaluated by comparing preoperative and postoperative scores on the Shorr blepharospasm grading scale, Burke–Fahn–Marsden Dystonia Rating Scale (BFMDRS-M), and Blepharospasm Disability Index (BSDI).

**Results:**

Based on the Shorr grading scale, preoperatively, 97.9% of patients were grade 4, whereas 2.1% were grade 3. Postoperatively, 87.5% achieved complete remission (grade 0), whereas 7.4, 3.7, 0.9, and 0.5% were grades 1, 2, 3, and 4, respectively. Pre- and postoperative BFMDRS-M scores significantly differed: eye (8.0 vs. 0.0), mouth (3.0 vs. 0.0), speech/swallowing (3.0 vs. 2.0), neck (3.0 vs. 1.5), and total (8.0 vs. 0.0). BSDI scores also significantly differed pre- and postoperatively. The results indicate that NCB effectively alleviates blepharospasm and addresses the lack of a definitive treatment, without causing severe local or systemic complications such as facial paralysis.

**Conclusion:**

NCB can safely and effectively alleviate blepharospasm, improve quality of life, and demonstrate stable long-term efficacy.

## 1 Introduction

Blepharospasm is a form of focal dystonia that often leads to functional blindness and severely impairs patients’ quality of life (1–3). The cause of the disorder remains unknown, and various pharmacological treatments have failed to achieve satisfactory therapeutic outcomes (4, 5). Surgical interventions, such as selective facial neurectomy, periocular muscle resection, thalamotomy, and pallidotomy (6), have also yielded unsatisfactory outcomes. Currently, when first-line botulinum toxin therapy fails, deep-brain stimulation (DBS) is considered the final treatment option (7). Although numerous small case series indicate the benefits of DBS,3 approximately 25% of patients are non-responders (8). The response of blepharospasm to DBS varies (9), and the level of evidence supporting DBS for MS remains limited (7). When treatments recommended by consensus statements and guidelines prove ineffective (10, 11), there remains a lack of targeted therapeutic strategies for blepharospasm (12). Through 13 years of clinical observation (13), we have demonstrated that blepharospasm is closely associated with hyperexcitability of the blink reflex induced by negative emotions. Using ocular surface electrophysiology, we demonstrated that blocking branches of the trigeminal nerve can alleviate lower eyelid spasm. Based on this, we developed a surgical technique termed neural circuit blockade (NCB) for treating blepharospasm. This method alleviates trigeminal nerve stimulation by facial expression muscles, decreasing the generation and transmission of sensory signals, attenuating feedback-driven and plasticity-related motor information functions, lowering the tension of the orbicularis oculi (14). Unlike previous approaches, NCB does not require resection of facial nerve branches, thereby avoiding complications such as facial paralysis. Additionally, in contrast to periorbital myectomy, it preserves the orbicularis oculi muscle and maintains normal structure and function. A total of 4,194 patients received NCB treatment. This study aimed to analyse the efficacy and safety of NCB in 570 patients with blepharospasm, with a median follow-up of 51 months.

## 2 Subjects and methods

### 2.1 Study design and participants

This single-centre, retrospective case series included 658 patients diagnosed with blepharospasm at the Meige Syndrome Centre of the Third People’s Hospital of Henan Province, China, between July 2019 and June 2022, who subsequently underwent NCB. The longest interval from surgery to follow-up was 6 years, with a median of 51 months. Because the patients were of advanced age and widely distributed geographically, in-person follow-up could not be conducted for all. Additionally, due to changes in contact information, address, health status, and other reasons, the rate of loss to follow-up was 13.37% (88/658). Ultimately, 570 patients were included in the analysis, reasonably representing the treatment outcomes of this cohort. This study strictly adheres to the STROBE guidelines to ensure data authenticity and accuracy (with a 95% confidence interval). The study protocol was approved by the Ethics Committee of the Third People’s Hospital of Henan Province on March 1, 2018 (approval number: 2018-KY-008). The study was conducted in accordance with the Declaration of Helsinki, and written informed consent was obtained from all patients. All surgical procedures were uniformly performed by three surgeons simultaneously under the supervision of a single senior physician.

The inclusion criteria were as follows: (1) patients presented with involuntary spasmodic contractions of the bilateral periorbital muscles, with or without oromandibular or cervical dystonia (15), and having undergone NCB surgery >38 months prior; (2) blepharospasm accompanied by mild or severe functional impairment (16); and (3) refractory to oral medications, botulinum toxin injections, and deep brain stimulation. The exclusion criteria comprised: (1) secondary MS due to central nervous system lesions; (2) somatic symptoms of psychiatric disorders, parkinsonian dyskinesia, essential tremor; and (3) incomplete data or loss to follow-up.

### 2.2 NCB procedure

**Figure 1:**
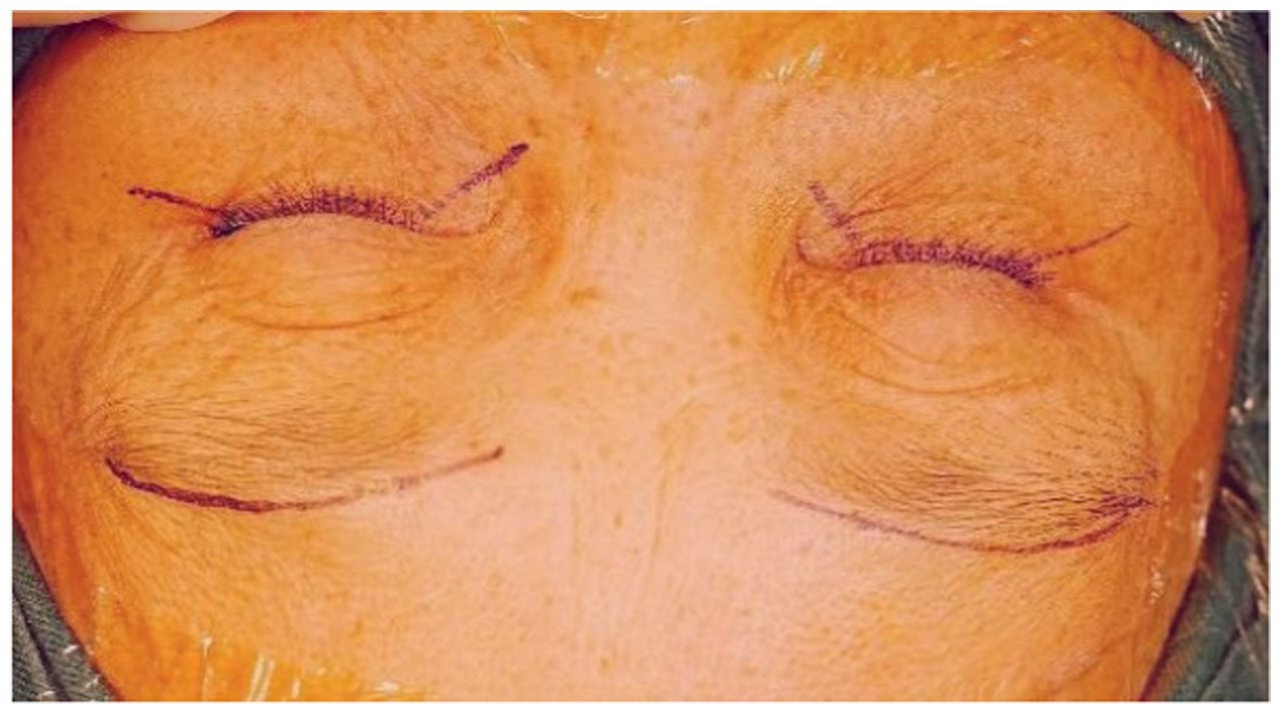
Draw lines along the superior margin of the eyebrow and the lower eyelid margin.

**Figure 2:**
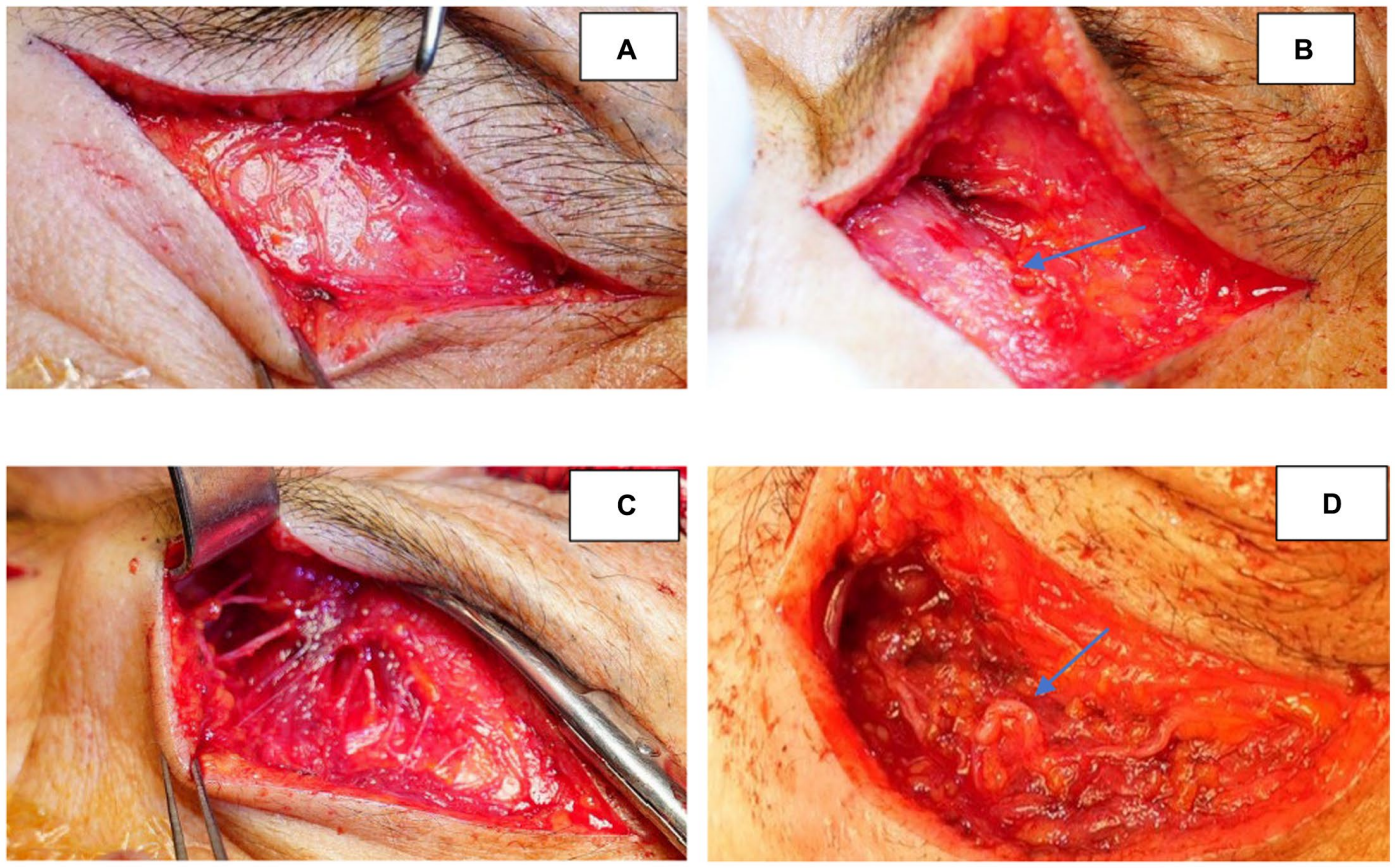
**(A)** Incision of the skin and orbicularis oculi muscle. **(B)** Exposure of the corrugator supercilii muscle (arrow). **(C)** Supraorbital neurovascular plexus within the corrugator supercilii muscle (preserved). **(D)** Tortuous branches of the supraorbital nerve coursing in the corrugator supercilii muscle (arrow).

**Figure 3:**
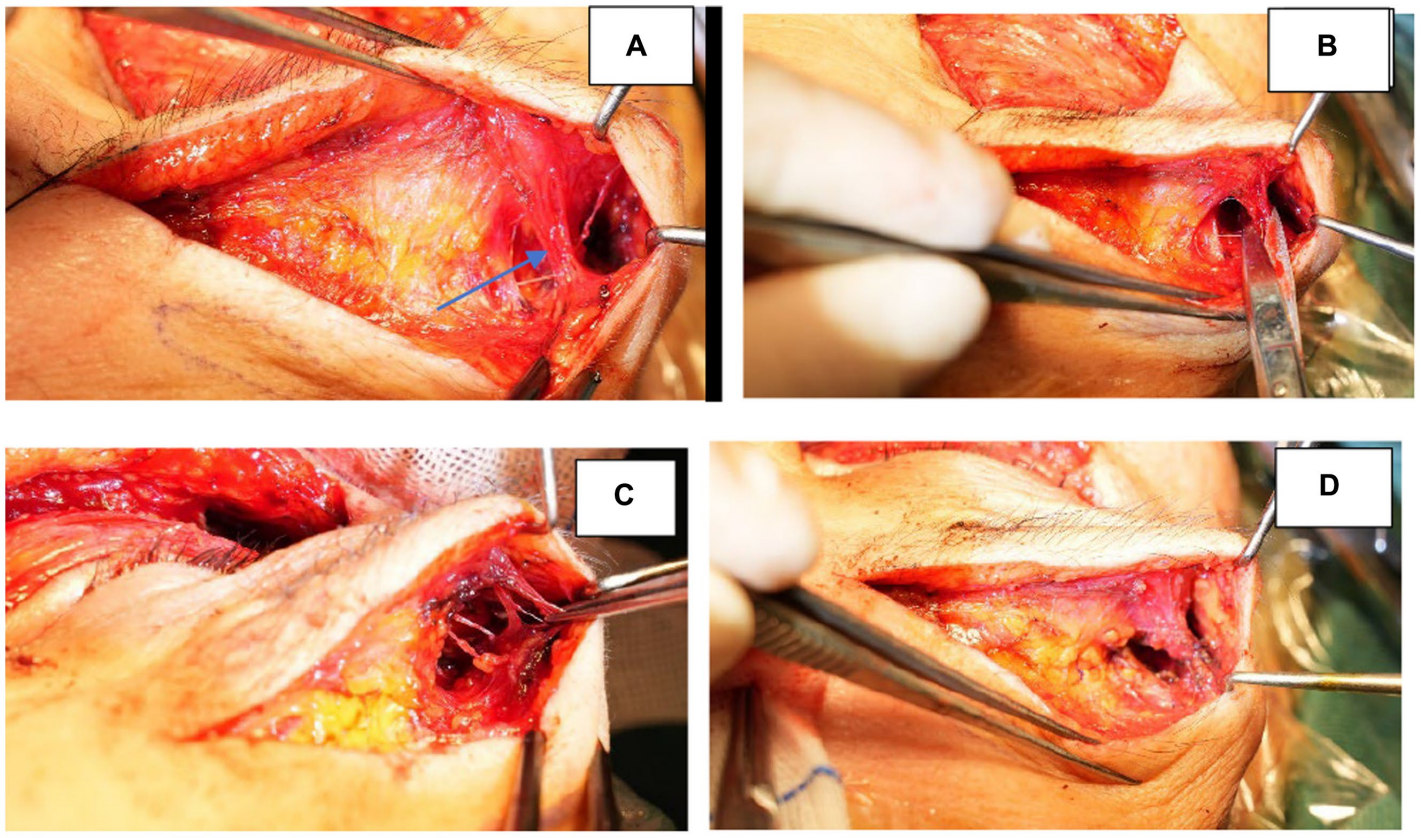
**(A)** Dissect the orbicularis oculi muscle temporally via the suprabrow incision. **(B)** Transect the orbicularis oculi muscle. **(C)** Identify and cut the nerve branches within the tunnel. **(D)** Replace and suture the orbicularis oculi muscle *in situ*.

**Figure 4:**
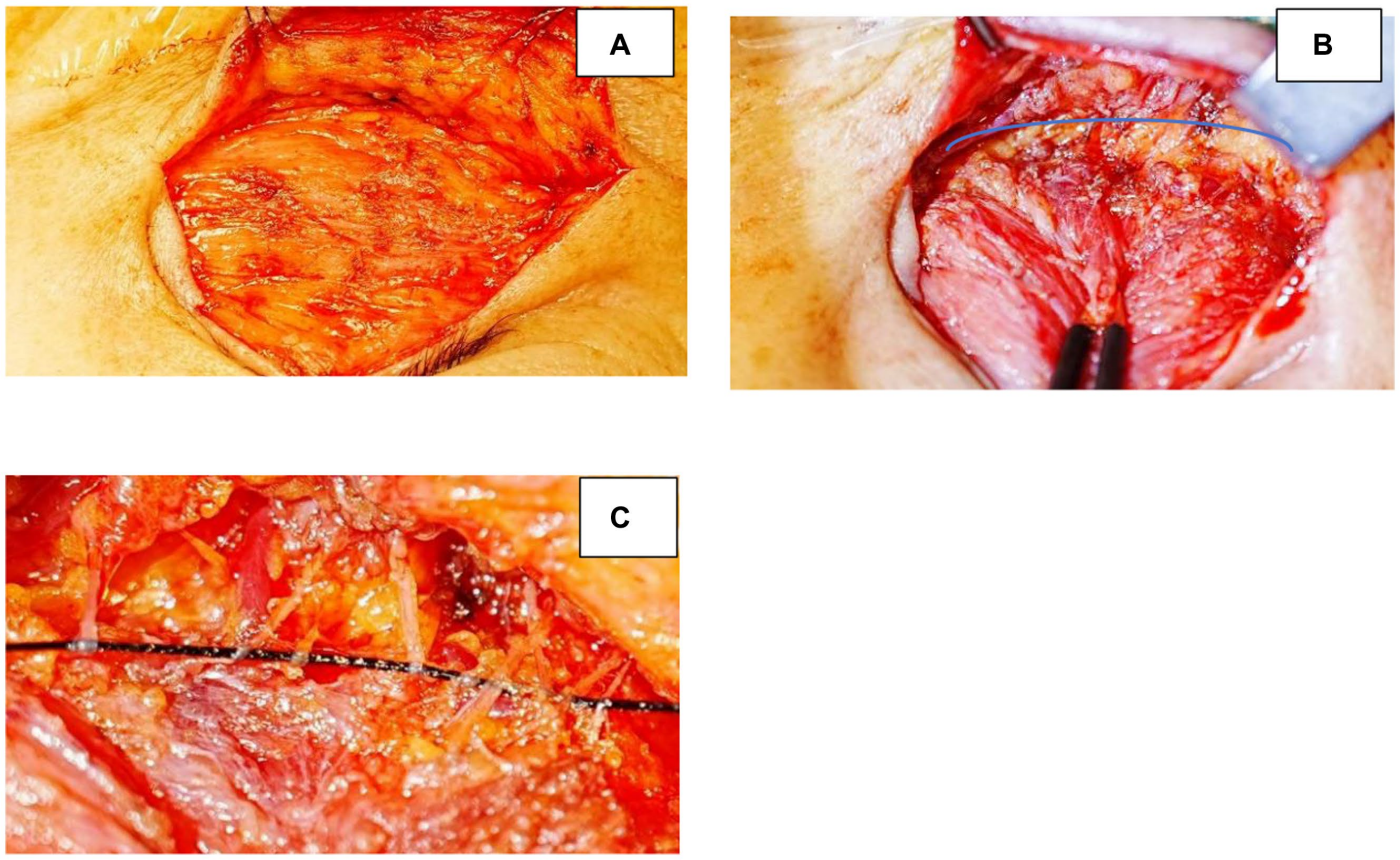
**(A)** Subcutaneous dissection of the lower eyelid extending to the orbital rim. **(B)** Incise the orbicularis oculi muscle at the inferior orbital rim (blue line). **(C)** Demonstrate the neural branches innervating the lower orbicularis oculi muscle, followed by transection.

**Figure 5:**
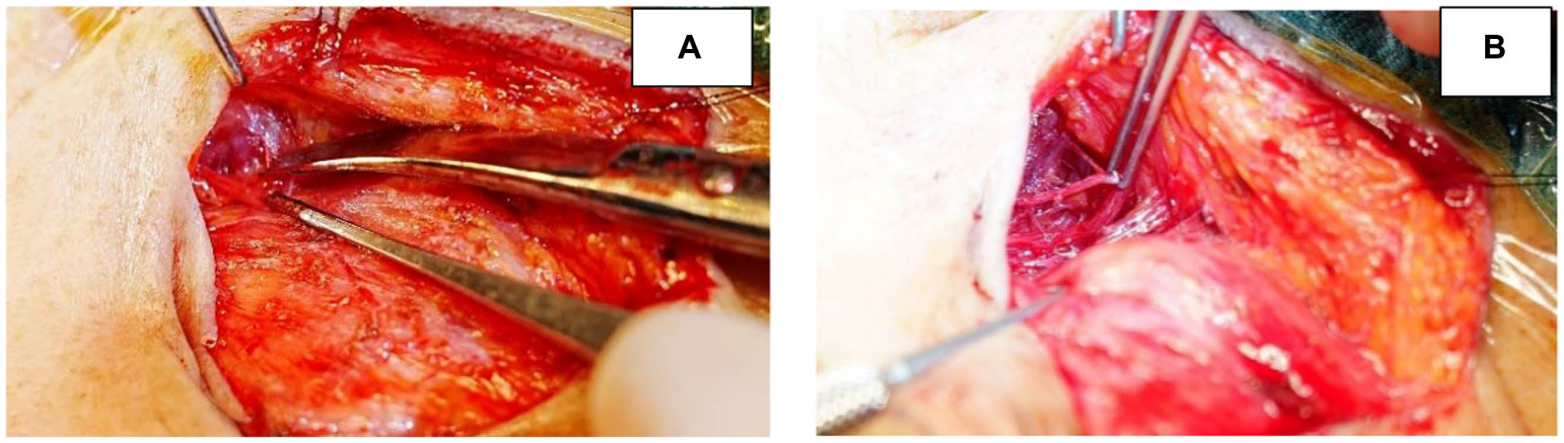
**(A)** The angular nerve is dissected within the levator labii superioris alaeque nasi muscle toward the medial canthus. **(B)** The angular nerve is demonstrated, which consists of the terminal branches of the zygomatic and temporal branches of the facial nerve, innervating the corrugator supercilii and procerus muscles.

**Figure 6:**
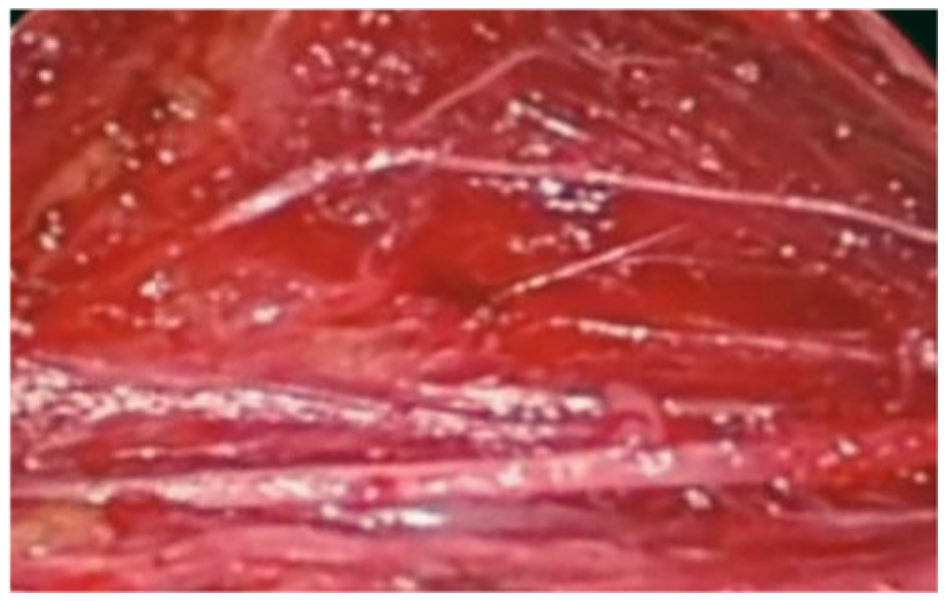
The orbicularis oculi muscle at the medial canthus is known as the “power source” of the orbicularis oculi. It is innervated by the zygomatic branch of the facial nerve. This nerve branch courses posterior to the orbicularis oculi muscle, extending from the temporal side all the way to the nasal side, and we term it the long deep zygomatic nerve.

**Figure 7:**
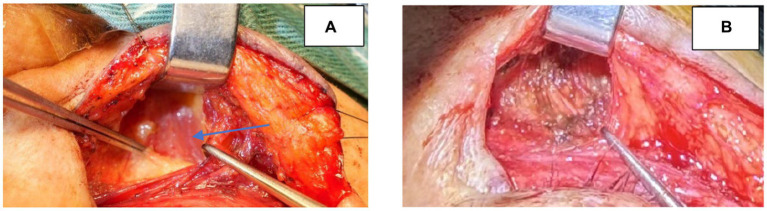
**(A)** Levator labii superioris overlying the infraorbital foramen. **(B)** Transection of the levator labii superioris reduces irritation to the infraorbital nerve.

**Figure 8:**
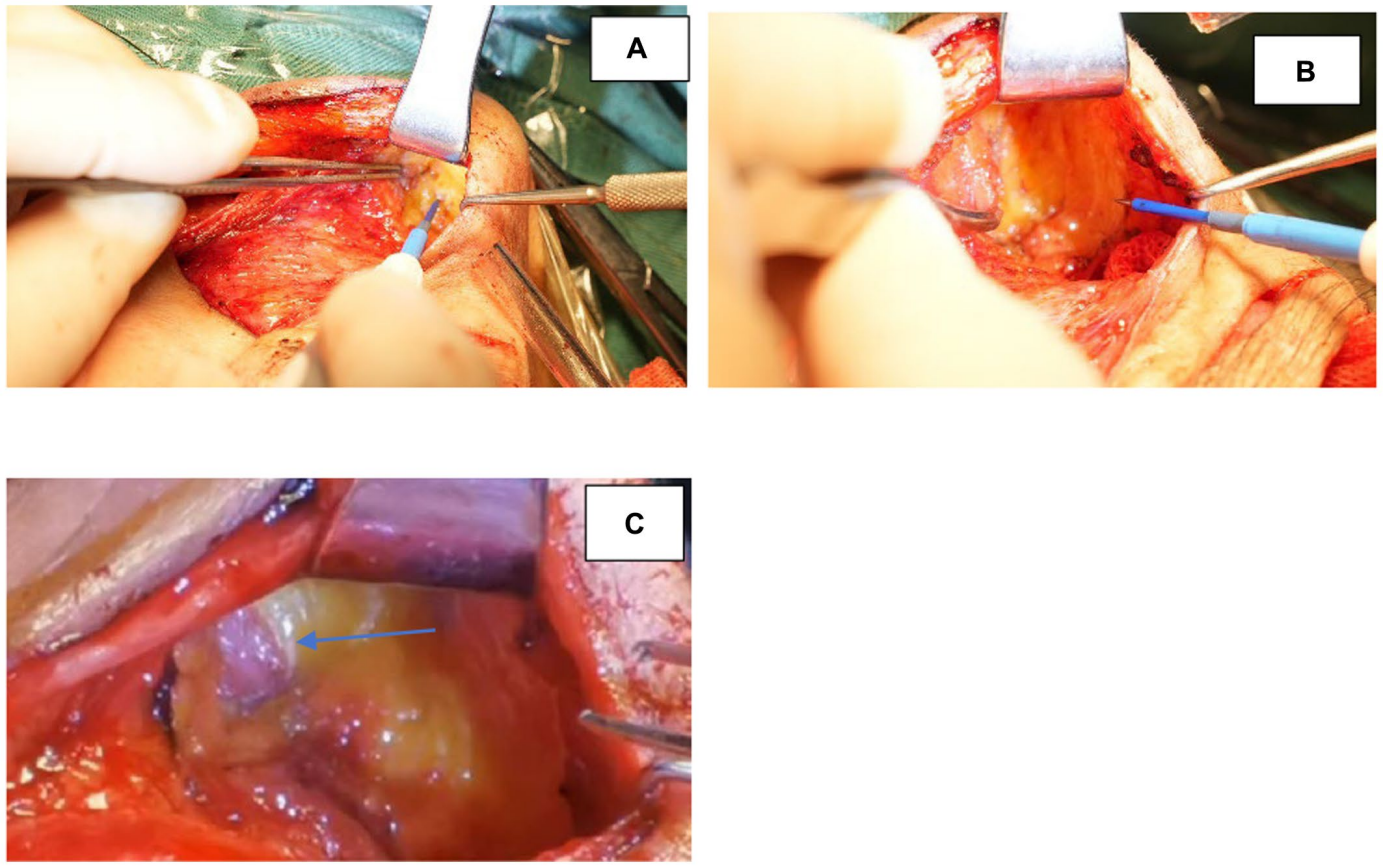
**(A)** Incise the fatty layer at the temporal side of the lower eyelid. **(B)** The subcutaneous tunnel extends deeply to the zygomatic arch. **(C)** Transect the zygomaticus major muscle (arrow).

**Figure 9:**
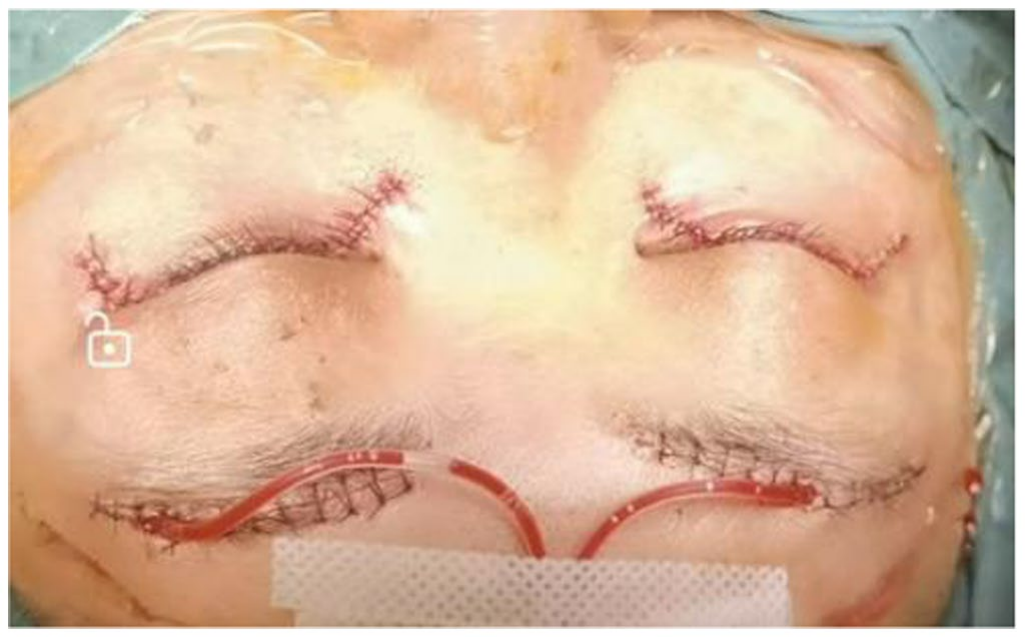
Postoperative face and incision.

**Figure 10:**
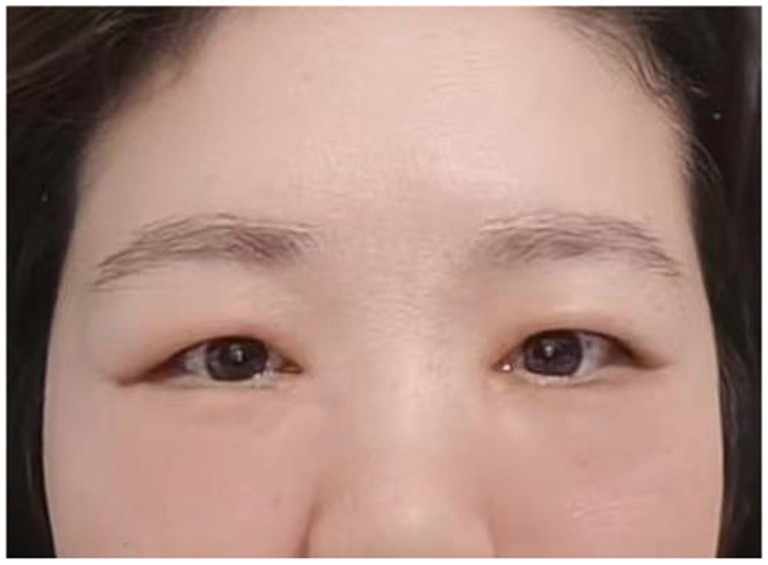
Images after NCB recovery.

The operation was performed under general anaesthesia as follows:

1. Methylene blue was used to mark the lines at the upper margin of the eyebrow and 2 mm below the lower eyelash line. The lower eyelid markings extended inferomedially by approximately 8 mm on the nasal side and inferotemporally by approximately 10 mm on the temporal side (Figure 1).
2. A skin incision was made along the superior brow line, dividing the skin and the orbicularis oculi muscle. ① The corrugator supercilii muscle was exposed by gentle superior and inferior dissection. Its muscle fibers were carefully excised, preserving the frontal branches that course perpendicular to the muscle fibers and the nerve branches running parallel to the fibers within the muscle; careful attention was also given to protecting the supraorbital nerve branches deep to the muscle and the veins deep to the corrugator insertion. Dissection was then continued medially, and the procerus insertion was resected under direct vision. The orbital portion of the orbicularis oculi muscle was not removed, in order to avoid impairing upper eyelid closure postoperatively (Figures 2A–D). ② On the temporal side of the incision, the orbicularis oculi muscle was dissected toward the lateral canthus, and the temporal branches of the facial nerve that supply the orbicularis oculi were blocked within a subcutaneous tunnel (Figures 3A–D).
3. A skin incision was made along the lower eyelid margin, and subcutaneous dissection was performed down to the infraorbital rim. ① The orbicularis oculi muscle was incised along the inferior orbital rim, and the branches of the neural network that supply the orbicularis oculi were blocked (17) (Figures 4A–C). ② At the nasal root, the “angular nerve” was sought within the levator alae nasi muscle and transected (18) (Figures 5A,B; Video 1). ③ Inferolateral to the medial canthal tendon, the “long deep zygomatic nerve” was identified deep to the orbicularis oculi muscle and blocked, in order to reduce muscle tone in the medial canthal portion of the orbicularis oculi (Figure 6; Video 2). ④ At the superior margin of the infraorbital foramen, a portion of the insertion of the levator labii superioris muscle was transected to eliminate irritation of the infraorbital nerve (Figures 7A,B). ⑤ On the temporal side of the lower lid incision, dissection was carried downward through the subcutaneous fat layer to expose the zygomaticus major and zygomaticus minor muscles, which were transected to relieve perioral spasm symptoms and to prevent downward progression of the periorbital symptoms (Figures 8A–C)
4. The cut ends of the zygomaticus major and zygomaticus minor muscles were anchored to the distal soft tissue to prevent localized contour depression. The orbicularis oculi muscle and associated soft tissues that had been divided along the inferior orbital rim were fixed to the periosteum of the infraorbital rim. The lower eyelid skin was reapproximated and closed with interrupted sutures. The supra-brow incision was closed in layers.
5. Negative pressure drains were placed lateral to both eyebrows, and a pressure dressing was applied (Figure 9). The drains were removed on the following day. Dressings were changed daily, and sutures were removed 1 week postoperatively. Postoperative recovery is shown in Figure 10.

### 2.3 Outcome assessment

The BFMDRS-M subscale (19) was used to assess eye, mouth, swallowing/speech, and neck symptom severity pre- and postoperatively. Using the BSDI (20) to evaluate the improvement in blepharospasm-related disability effectively reflects the improvement in patients’ quality of life. Additionally, the Shorr Blepharospasm Grading Scale (16) was used to assess pre- and postoperative changes in blepharospasm severity; this scale is simple, clear, and widely accepted. In a previous study, we observed ocular surface electrophysiological changes in 412 patients with Meige syndrome and 102 normal controls, and classified blepharospasm into four grades based on the amplitude and morphology of the electrophysiological waves, and into four types according to the conditions and timing of spasm wave occurrence (21). However, because a universally accepted standard is lacking, the severity of blepharospasm was still graded using the Shorr grading scale. The improvement in spasm severity was expressed as the percentage reduction in the BFMDRS score, calculated by subtracting the score at the last follow-up from the preoperative score, dividing the result by the preoperative score, and multiplying by 100%.

### 2.4 Statistical analysis

Statistical analyses were performed using SPSS software (version 21.0). The normality of the data distribution was tested using the Kolmogorov–Smirnov test. A *p*-value <0.05 indicated a non-normal distribution. Non-normally distributed data were presented as medians (Q1 and Q3). The Wilcoxon signed-rank test was used for paired comparisons, whereas the Kruskal–Wallis test was used for between-group comparisons. Categorical data were expressed as frequencies, percentages, or proportions. All tests were two-sided, with a p-value <0.05 considered statistically significant. Age, preoperative disease duration, postoperative follow-up duration, blepharospasm grading scores, degree of ocular spasm improvement, preoperative BSDI score, and postoperative BSDI score were non-normally distributed.

## 3 Results

In total, 570 patients were included. Of these, 189 were men (33.2%), and 381 were women (66.8%). The median age was 57 years (interquartile range [IQR]: 51–64 years; range: 19–79 years). The median preoperative disease duration was 3.0 years (IQR: 2.0–6.0 years; range: 0.5–35.0 years), and the median follow-up period was 51 months (IQR: 43–60 months; range: 38–72 months). Based on subtype classification: 323 patients (56.7%) had blepharospasm, 175 patients (30.7%) had blepharospasm and oromandibular dystonia, and 72 patients (12.6%) had blepharospasm combined with oromandibular and spasmodic torticollis. Baseline BFMDRS-M, BSDI, and blepharospasm grading scores are presented in Table 1. Previous treatments included dry eye therapy (76.14%), botulinum toxin injections (68.42%), traditional Chinese herbal medicine (34.91%), antipsychotic medications (28.42%), acupuncture (27.72%), and various surgical procedures, including stereotactic lesioning and DBS (18.50%). Each patient may have undergone multiple treatments.

**Table 1:** Demographic and baseline characteristics of patients.

Characteristic	Overall (*N* = 570)
Women, *n* (%)	381 (66.8%)
Age, years	57 (51, 64)
Disease duration, years	3.0 (2.0, 6.0)
Follow-up duration, months	51 (43, 60)
Classifications	
Blepharospasm	323 (56.7%)
Blepharospasm-oromandibular dystonia	175 (30.7%)
Blepharospasm-oromandibular dystonia combined with spasmodic torticollis	72 (12.6%)
BFMDRS-M	
Eye	8.0 (8.0, 8.0)
Mouth	3.0 (1.5, 4.5)
Speech/swallowing	3.0 (1.8, 6.0)
Neck	3.0 (1.0, 6.0)
Total	8.0 (8.0, 11.0)
BSDI	18.0 (15.0, 18.0)
Shorr blepharospasm grading scale	
Grade 3	10 (1.75%)
Grade 4	560 (98.25%)

Values are presented as median (Q1, Q3), or n (%). BDSI = Blepharospasm Disability Index, BFMDRS-M = Burke–Fahn–Marsden Dystonia Rating Scale-Movement.

The preoperative and postoperative BFMDRS scores and improvement rates are presented in Table 2. The total median (Q1, Q3) BFMDRS-M pre and postoperative score was 8.0 (8.0, 11.0) and 0.0 (0.0, 1.0), respectively, with the difference being statistically significant (*p* < 0.001). The overall improvement rate was 100% (IQR: 91.7, 100%). The median BFMDRS-M subscale scores for the eye, mouth, speech/swallowing, and neck were also significantly lower postoperatively than those observed preoperatively (all p < 0.001), with improvement rates of 100, 100, 12.5, and 62.5%, respectively. The median pre- and postoperative BSDI score also differed significantly (18 [15, 18] vs. 0 [0, 0], *p* < 0.001).

**Table 2:** Preoperative and postoperative BFMDRS and BSDI scores with corresponding improvement rates.

Outcome	Preoperative	Postoperative	*Z*-value	*p*-value	Improvement rate
Eye	8.0 (8.0, 8.0)	0.0 (0.0, 0.0)	−22.41	<0.001	100.0 (100.0, 100.0)
Mouth^a^	3.0 (1.5, 4.5)	0.0 (0.0, 2.0)	−12.59	<0.001	100.0 (50.0, 100.0)
Speech/swallowing^b^	3.0 (1.8, 6.0)	2.0 (0.0, 4.0)	−4.15	<0.001	12.5 (0.0, 100.0)
Neck	3.0 (1.0, 6.0)	1.5 (0.0, 3.0)	−4.48	<0.001	62.5 (0.0, 100.0)
Total^c^	8.0 (8.0, 11.0)	0.0 (0.0, 1.0)	−21.08	<0.001	100.0 (91.7, 100.0)
BSDI	18.0 (15.0, 18.0)	0.0 (0.0, 0.0)	−20.84	<0.001	100.0 (100.0, 100.0)

Values are presented as median (Q1, Q3). Differences between pre- and postoperative scores were analysed using the Wilcoxon signed-rank test. ^a^One patient scored 1 vs. 1.5 and another 0 vs. 1.5 (pre vs postoperative). ^b^One patient scored 3 vs. 6 and another 2 vs. 3, with worsened symptoms (pre vs postoperative). ^c^One patient scored 11 vs. 12.5 (pre vs postoperative). BFMDRS-M = Burke–Fahn–Marsden Dystonia Rating Scale-Movement; BSDI = Blepharospasm Disability Index.

**Figure 11:**
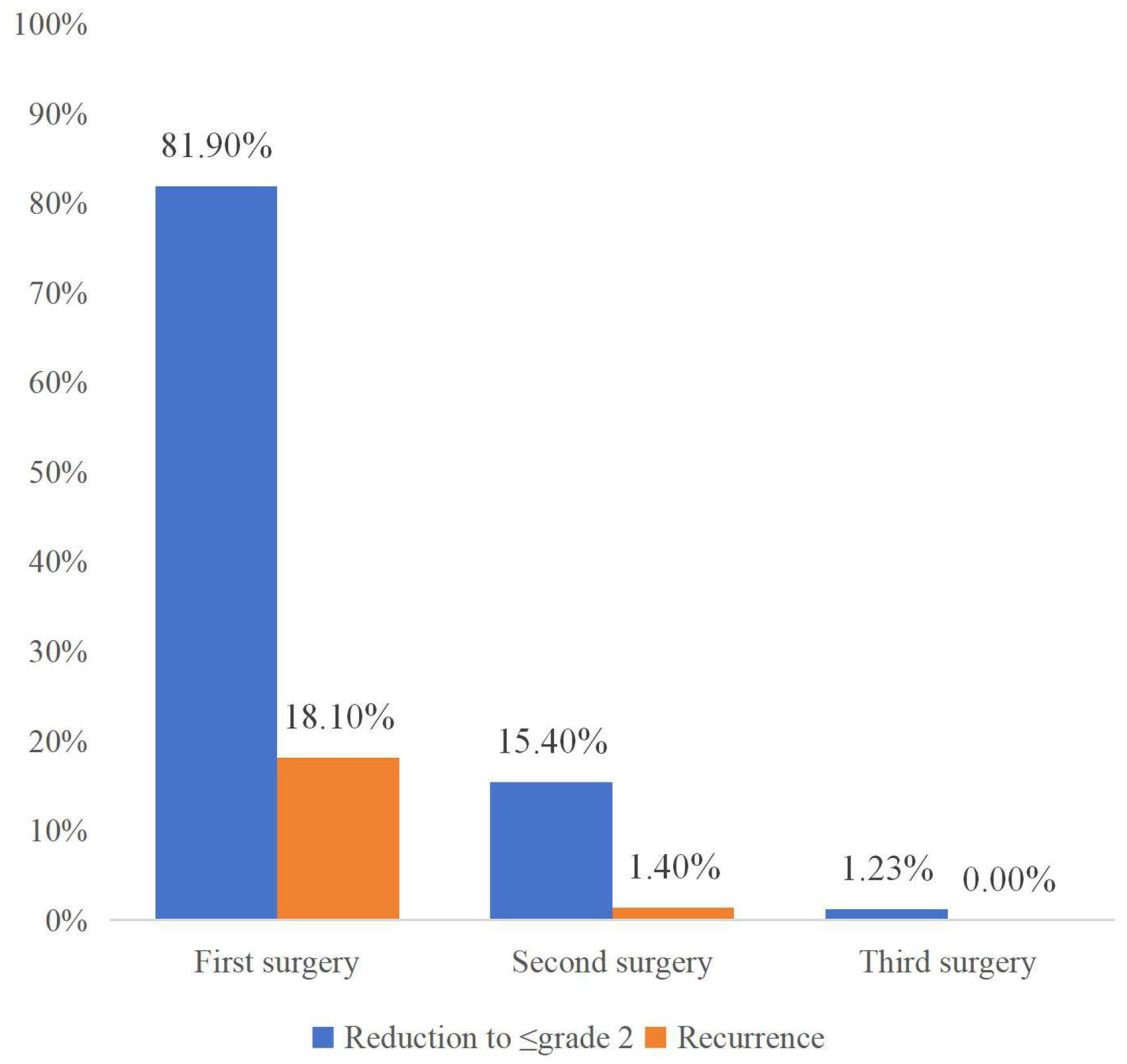
Number of patients with reduction to ≤grade 2 and recurrence.

**Figure 12:**
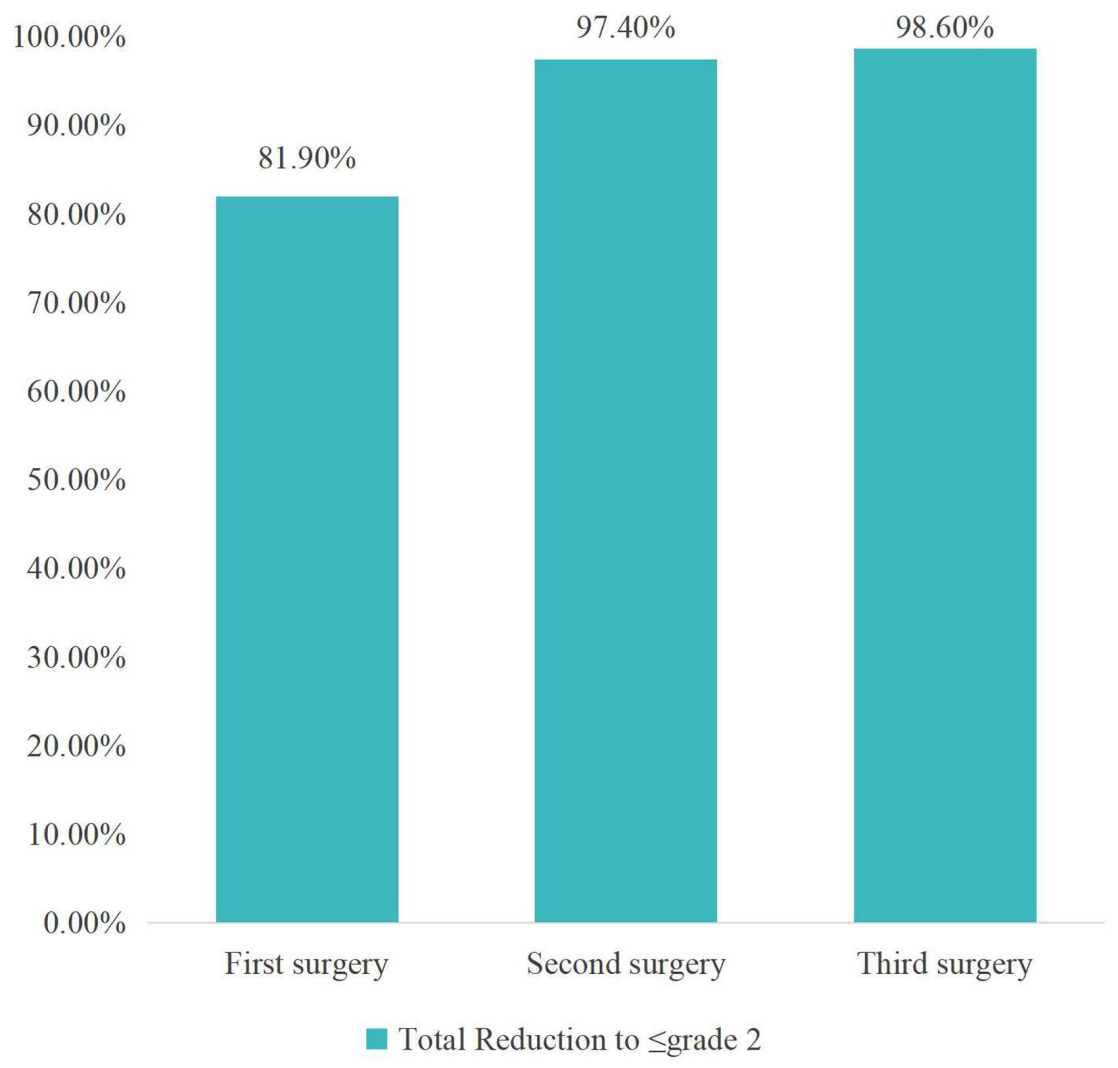
Total number of patients with reduction to ≤grade 2.

Preoperatively, 10 patients (1.75%) were classified as grade 3 on the Shorr blepharospasm grading, whereas 560 patients (98.25%) were grade 4. Postoperatively, 499 patients (87.5%) were grade 0, 42 (7.4%) were grade 1, 21 (3.7%) were grade 2, five (0.9%) were grade 3, and three (0.5%) were grade 4. Table 3 summarizes the reduction in blepharospasm to ≤grade 2 after each surgery and the recurrence rate. After the first surgery, blepharospasm decreased to ≤grade 2 in 467 of 570 patients (81.93%), with recurrence in 103 of 570 patients (18.1%) (seven patients discontinued treatment). After the second surgery (*n* = 96), blepharospasm decreased to ≤grade 2 in 555 of 570 patients (97.37%), with recurrence in eight out of 570 patients (1.40%) (one patient discontinued treatment). After the third surgery (*n* = 7), blepharospasm was ≤grade 2 in 562 of 570 patients (98.60%)(Figures 11, 12). No statistically significant difference was observed in postoperative BFMDRS-M eye score (*p* = 0.17) among blepharospasm subtypes (Table 4).

**Table 3:** Number of patients achieving blepharospasm grade ≤2 postoperatively and number of patients with blepharospasm recurrence.

Surgery order	Number of patients with reduction to ≤grade 2	Number of patients with recurrence	Total number of patients with reduction to ≤grade 2
First surgery (*n* = 570)	467/570 (81.93%)	103/570 (18.1%)	467/570 (81.93%)
Second surgery (*n* = 96)	88/570 (15.44%)	8/570 (1.40%)	555/570 (97.37%)
Third surgery (*n* = 7)	7/570 (1.23%)	0/570 (0.00%)	562/570 (98.60%)

**Table 4:** Comparison of pre- and postoperative BFMDRS-M eye and BSDI scores among blepharospasm subtypes.

Outcome	Blepharospasm	Blepharospasm-oromandibular dystonia	Blepharospasm-oromandibular dystonia combined with torticollis	*p*-value
Preoperative BFMDRS-M eye score	8.0 (8.0, 8.0)	8.0 (8.0, 8.0)	8.0 (8.0, 8.0)	0.02
Postoperative BFMDRS-M eye score	0.0 (0.0, 0.0)	0.0 (0.0, 0.0)	0.0 (0.0, 0.0)	0.17
Preoperative BSDI	18.0 (15.0, 18.0)	17.0 (15.0, 18.0)	18.0 (15.0, 18.0)	0.04
Postoperative BSDI	0.0 (0.0, 0.0)	0.0 (0.0, 0.0)	0.0 (0.0, 0.0)	0.01

Values are presented as median (Q1, Q3). Significant differences were analysed using the Kruskal–Wallis test. BDSI = Blepharospasm Disability Index, BFMDRS-M = Burke–Fahn–Marsden Dystonia Rating Scale-Movement.

**Figure 13:**
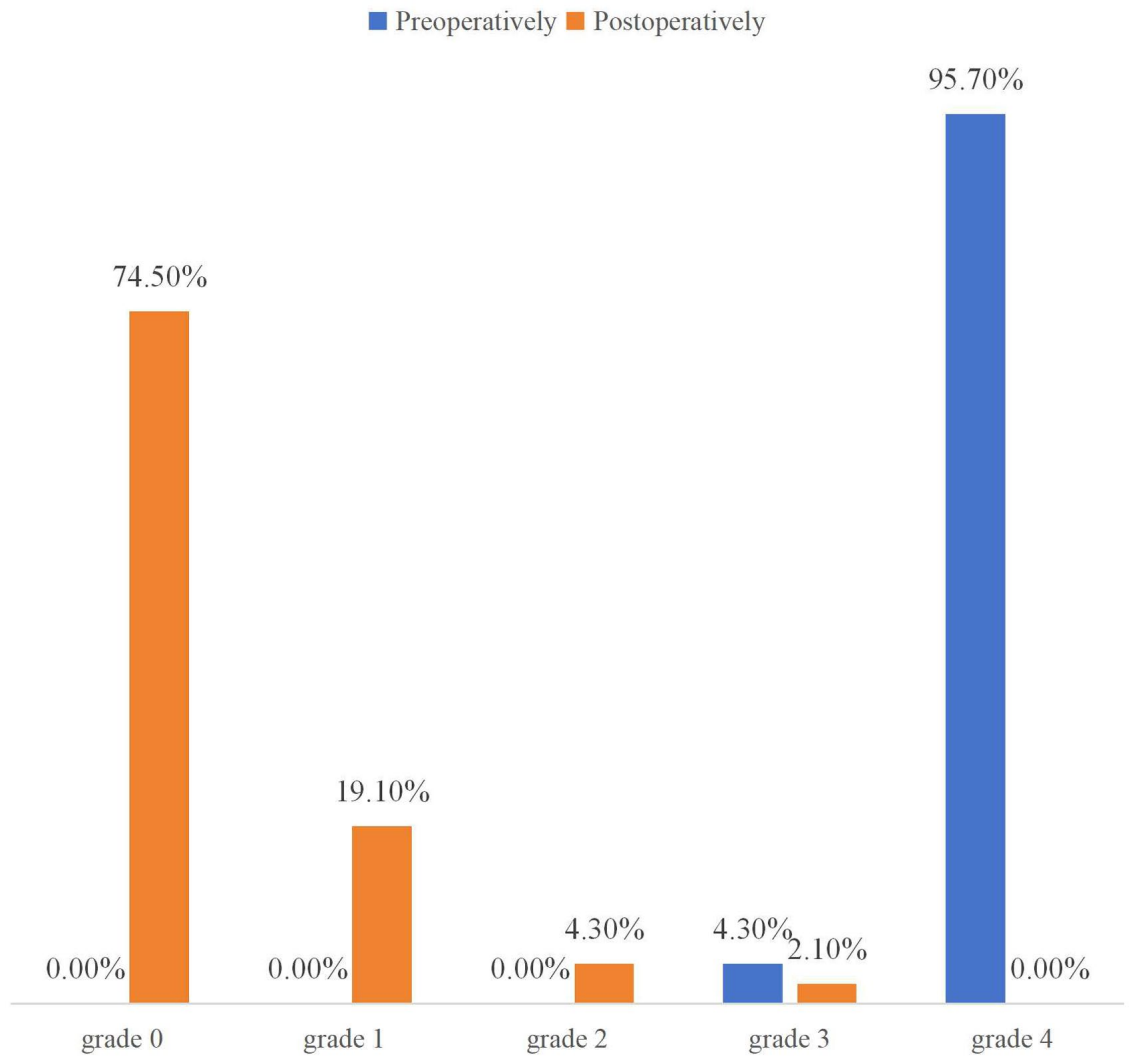
Preoperative and postoperative changes in blepharospasm grading in 47 patients with ineffective DBS treatment.

In this study, 47 patients showed no response to DBS. Preoperatively, 2 patients (4.3%) had grade 3 blepharospasm and 45 patients (95.7%) had grade 4 blepharospasm. Postoperatively, 35 patients (74.5%) achieved grade 0, 9 patients (19.1%) grade 1, 2 patients (4.3%) grade 2, and 1 patient (2.1%) grade 3 (Figure 13). The preoperative and postoperative BFMDRS eye subscores were [8.0 (8.0, 8.0) vs. 0.0 (0.0, 0.5)], and the total BFMDRS scores were [12.0 (10.0, 15.0) vs. 1.0 (0.0, 3.0)]. The postoperative BFMDRS mouth subscores were also significantly reduced (*p* < 0.05).

Comparison of BFMDRS improvement rates after GPi-DBS, DBS → NCB, and NCB treatment is shown in Table 5.

**Table 5:** Comparison of BFMDRS improvement rates after GPi-DBS, DBS → NCB and NCB treatment.

Variables	GPi-DBS (*n* = 36)	DBS → NCB (IQR) (*n* = 47)	NCB (IQR) (*n* = 570)
Follow-up time (months)	6; 12	20 (9, 41)	51 (43, 60)
BFMDRS improvement Rate (%)	66.9 ± 13.8%; 70.8 ± 21.0%	100.0% (91.7, 100.0%)	100.0% (100, 100.0%)

The results of GPi-DBS were reported by Ryoong Huh. DBS → NCB refers to NCB treatment administered to patients who failed to respond to DBS therapy. NCB represents the overall improvement rate of some patients after 2–3 operations.

Regarding NCB-related adverse events, forehead numbness was commonly observed. This was related to injury of the frontal branch of the supraorbital nerve. After preserving the frontal branch, the extent of numbness is reduced, with recovery typically taking 3–6 months, though a few cases may require over 12 months. Epiphora occurred in 48 of the 570 patients (80.00%) in the early postoperative period. It was related to local oedema, poor lower eyelid tension, and impaired drainage dynamics. The tearing gradually subsided as the oedema resolved and tension recovered. In a few cases, persistent long-term tearing was related to pre-existing poor drainage function or injury to orbicularis oculi function around the lacrimal sac. Lower eyelid ectropion occurred in 48 of the 570 patients (8.42%). It was associated with age, decreased lower eyelid tension postoperatively, and inadequate tissue repositioning or traction caused by adhesions during the healing period. In subsequent surgeries, attention is paid to proper tissue repositioning. When preoperative evaluation indicated low tension, intraoperative V-shaped excision of the lower eyelid was performed, along with measures to reduce tension during the healing process. Consequently, postoperative ectropion has become rare. If ectropion persisted for over a year, surgical correction was required. A decrease in eyelid closing force occurred in 30 of the 570 patients (5.26%). In these cases, the cornea was not exposed during sleep, and corneal function was unaffected. At this time, care should be taken to prevent foreign objects (such as water) from entering the eye. Lower eyelid retraction occurred in five of the 570 patients (0.87%), which was associated with traction from adhesions during healing and compensatory hyperfunction of Müller’s muscle secondary to decreased orbicularis oculi muscle tone. Lateral canthoplasty or Müller’s muscle resection may be performed when necessary. Lower eyelid entropion occurred in two of the 570 patients (0.35%), related to alterations in lower eyelid tension, and was corrected surgically (Table 6).

**Table 6:** Surgery-related complications.

Complications	Cause	Management
Forehead numbness (*n* = 570)	Injury to the frontal branch of the supraorbital nerve	Protect the nerve branch; allow for natural recovery
Early tearing (*n* = 456)	Eyelid oedema, hypotonia, or injury of the orbicularis oculi muscle	Wait for oedema to subside and muscle tone to recover
Lower eyelid ectropion (*n* = 51)	Eyelid laxity, decreased tension, inadequate repositioning, scar adhesion traction	Ensure proper tissue repositioning, increase lower eyelid tension, postoperative correction
Decreased eyelid closure strength (*n* = 30)	Hypotonia of the orbicularis oculi muscle	Protect the cornea; prevent foreign body entry
Eyelid retraction (*n* = 5)	Scar adhesion traction, hyperactivity of Muller’s muscle	Tension-reduced healing, resection of Muller’s muscle
Lower eyelid entropion (*n* = 2)	Associated with lower eyelid tension	Surgical correction

## 4 Discussion

In this retrospective study of patients with blepharospasm, NCB effectively alleviated blepharospasm, demonstrating long-term efficacy and safety. This is a surgical treatment aimed at ultimately relieving blepharospasm. After the first surgery, blepharospasm was reduced from grade 3–4 to ≤2 in 81.93% of patients. Among the 18.07% with residual or recurrent spasms, 96 out of 103 cases (93.20%) underwent a second surgery, and of these, 7 patients received a third surgery after two years, with blepharospasm reduced to ≤2. In this study of 570 patients,the BFMDRS-M eye score pre- and postoperatively significantly improved from 8.0 to 0.0, with an improvement rate of 100.0%. The BSDI score also decreased significantly from 8 preoperatively to 0 postoperatively, indicating improved quality of life.

The sustained long-term efficacy of NCB can be attributed to prolonged observation and in-depth understanding of blepharospasm. The blink reflex circuit comprises afferent signals carried by the trigeminal nerve, efferent signals via the facial nerve, and the orbicularis oculi serving as the final effector. Sudden adverse events (22) and long-term mental stress (23) cause spastic contractions of the corrugator supercilii, which forms vertical glabellar lines while also stimulating the supraorbital nerve at the afferent end of the blink reflex, leading to hyperactive blink reflex and frequent blinking (24). Overflow activation accompanied by hyperfunction of the blink reflex generalizes the blink function of the palpebral orbicularis oculi to the orbital orbicularis oculi (25), resulting in difficulty in eye opening. The organic integration of psychological factors with blepharospasm via the facial expression muscle-blink reflex pathway has deepened the understanding of the inducing mechanism of blepharospasm (26). Persistent spasm of the corrugator supercilii muscle, coupled with reflex motor output in response to abnormal sensory input, transforms the blink reflex circuit into a pathological pathway that induces blepharospasm (27).

**Figure 14:**
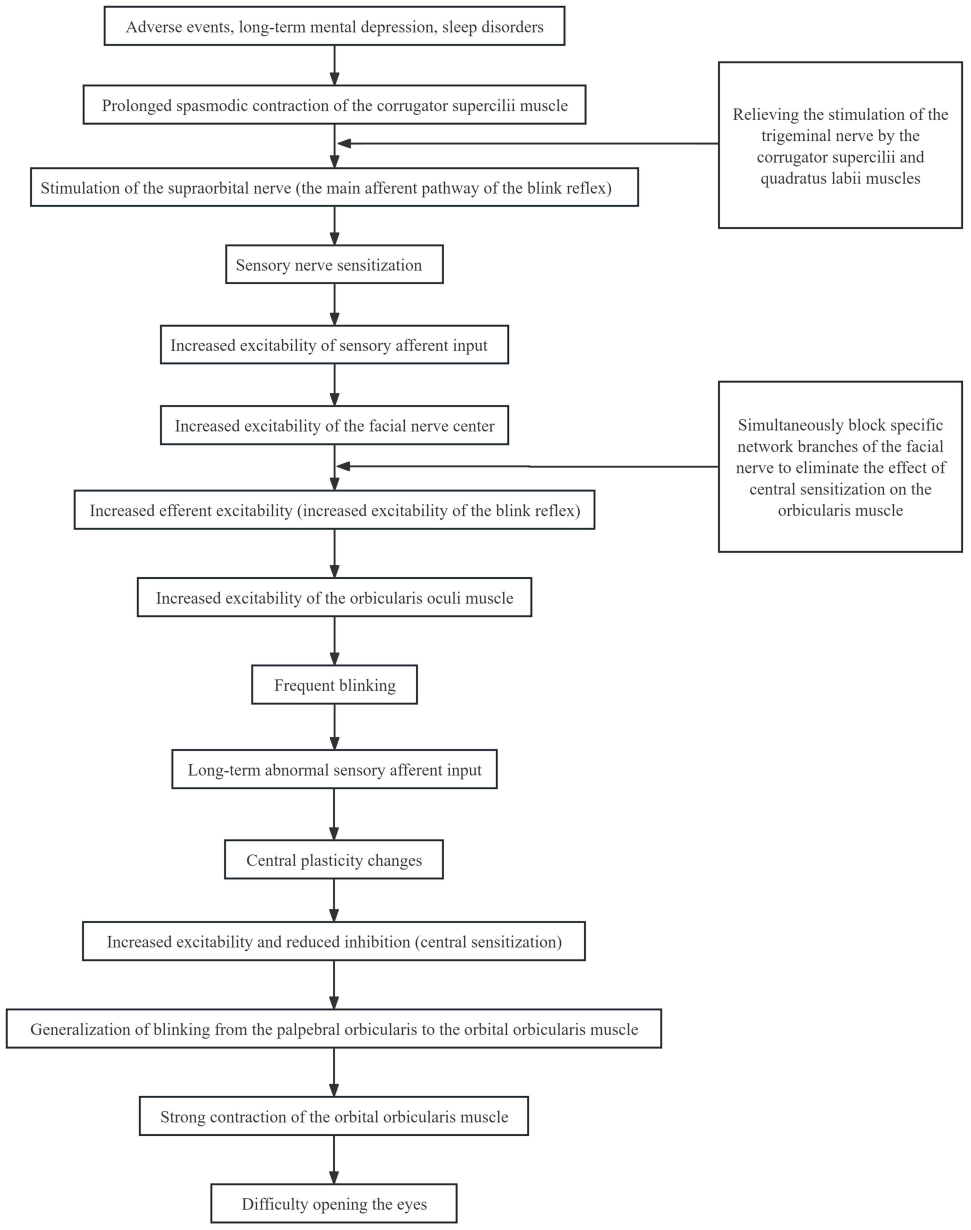
Schematic roadmap of neural circuit blockade.

When conducting surface electrophysiological examinations for blepharospasm (21), we observed that spasm waves of the inferior palpebral orbicularis oculi muscle were attenuated in a small number of patients after corneal anaesthesia of the conjunctival sac with 0.5% proparacaine eye drops. Blockade of the supraorbital or infraorbital nerve with 2% lidocaine relieved inferior eyelid spasm in some patients, further confirming the correlation between abnormal sensory input and blepharospasm. Based on these findings, we developed a therapeutic regimen termed NCB, which is designed to eliminate the stimulatory effect of the corrugator supercilii and levator labii superioris muscles on the trigeminal nerve, reduce abnormal sensory input, attenuate reflex motor output, and block specific reticular branches of part of the facial nerve circuit to weaken the influence of central plasticity on the orbicularis oculi muscle. Schematic roadmap of neural circuit blockade is shown in Figure 14.

The concept of NCB is inspired by the ‘sensory trick’, the most distinctive and characteristic clinical sign of MS. The sensory trick refers to specific sensory stimuli that temporarily alleviate spasms, indicating that the core pathological mechanism of MS lies in the dysfunction of the sensorimotor integration circuit. The gating function of the basal ganglia, which normally filters sensory information, is impaired. This leads to abnormal amplification (sensitisation) of subthreshold normal sensory inputs—such as ocular sensations—thereby triggering excessive motor responses. The triggering mechanism of blepharospasm is primarily related to the stimulation of the trigeminal nerve by facial expression muscles. A new approach to treating blepharospasm has gradually taken shape, which weakens motor information by removing sensory input, thereby promoting the restoration of normal sensorimotor loop function.

The branches of the facial nerve intercommunicate and fuse during their course to the orbicularis oculi (17, 18), forming a three-dimensional neural network for its innervation. The compensatory function of the neural network ensures that, after specific branches are blocked, normal innervation of the orbicularis oculi is preserved, thereby maintaining proper contractile function.

Facial nerve avulsion and periorbital myectomy were traditionally the optimal methods for relieving blepharospasm. Facial nerve avulsion involves cutting the branches innervating the orbicularis oculi at their exit from the parotid gland and avulsing them from the muscle. However, severe facial palsy complications and a high recurrence rate (22/27 within 1 year) led to its gradual abandonment (28). In 1951, Fox proposed a new method for resecting the upper and lower orbicularis, while preserving the pretarsal fibres, though its spasm-relieving effects were limited. Three decades later, Gillum and Anderson modified the procedure of Fox by completely resecting the orbicularis oculi, corrugator supercilii, depressor supercilii, and procerus muscles. However, owing to severe lymphedema, they subsequently recommended limiting the surgery to the upper eyelids. Approximately 20–30% of patients still require subsequent resection of the lower orbicularis oculi (28). Many surgeons have observed that, despite meticulous technique, complete excision of the orbicularis muscle is unattainable, contributing to blepharospasm recurrence. Postoperatively, botulinum toxin injections are still necessary, although the required dosage is lower than that of previous treatments. NCB can precisely block specific branches of the neural network without causing side effects such as facial paralysis. It eliminates the need to resect the orbicularis oculi, thereby preserving the normal structure and function of the eyelid. The lower eyelid serves as the primary pathway for the zygomatic and buccal branches of the facial nerve, which innervate the periocular muscles. By blocking the ‘angular nerve’ in the lower eyelid, spasms of the corrugator supercilii and procerus are relieved. Blocking the ‘long deep zygomatic nerve’ reduces tension in the medial canthal portion of the orbicularis oculi, whereas blocking the network branches that innervate the lower orbicularis oculi alleviates spasms in this region. In addition, tendon release of the levator labii superioris alaeque nasi and levator labii superioris eliminates stimulation of the infraorbital nerve. Thus, the lower eyelid is an essential and indispensable component of NCB. The therapeutic strategy of NCB likely aligns closely with the pathological process of blepharospasm, offering more precise targeting, more reliable efficacy, and greater safety. The alleviation of symptoms such as dysarthria and upper limb tremors, which are distant from the surgical site, further supports the hypothesis that sensory nerve sensitisation and sensorimotor integration dysfunction are key mechanisms underlying MS (29).

In this cohort treated with NCB (*N* = 570), the median follow-up period was 51 months. The improvement rate for the ocular sub-score of the BFMDRS-M was 100.0% (IQR: 100.0, 100.0%), whereas that for the total score was 100% (IQR: 91.7, 100.0%). No severe postoperative complications were observed. In contrast, Ryoong Huh reported that GPi-DBS (N = 36) achieved a 1-year improvement rate of 70.8 ± 21.0% for the ocular subs-core and 60.7% for the total score, with intracranial haemorrhage occurring in four out of 36 cases (11%) (30). Regarding long-term efficacy, NCB demonstrates higher improvement rates and greater safety than GPi-DBS.

The limitations of this study are that it is a single-center retrospective case series. The patients came from all over China, were geographically dispersed, and represented a special population. Not all follow-up visits could be conducted face-to-face, resulting in a high loss to follow-up. Follow-up was performed via online video or telephone, which introduced potential bias in the data. The lack of blinded outcome assessment is a weakness of this study. We tried to present the full picture of the study as comprehensively as possible and did not exclude patients lost to follow-up; since this was not a pragmatic exclusion, it does not affect the study results. Although NCB has little impact on appearance, it is after all an injurious treatment and is only suitable for patients whose normal life is severely affected. It is not appropriate for patients without significant functional impairment, which limits its generalizability. Postoperatively, recovery from periorbital tightness may take one year or longer, and overflow symptoms associated with blinking, such as the movements of the orbicularis oculi and quadratus labii muscles that accompany blinking, cannot be completely eliminated.

## Data Availability

The datasets presented in this article are not readily available because the dataset contains confidential patient information and cannot be made publicly available due to ethical restrictions. Data are available from the corresponding author upon reasonable request and with permission of the ethics committee. Requests to access the datasets should be directed to Xianzhong Liu, 1776839026@qq.com.
